# Pain control due to botulinum toxin therapy in cervical dystonia relates to the sensorimotor integration process

**DOI:** 10.3389/dyst.2023.11362

**Published:** 2023-08-06

**Authors:** Aparna Wagle Shukla, Robert Chen, Wei Hu

**Affiliations:** 1 Department of Neurology, Fixel Institute for Neurological Diseases, University of Florida, Gainesville, FL, United States,; 2 University Health Network, Toronto Western Hospital, University of Toronto, Toronto, ON, Canada

**Keywords:** botulinum toxin, dystonia, cervical dystonia, sensorimotor integration, pain

## Abstract

**Background:**

Botulinum toxin (BoNT) injections have been found to improve pain symptoms of isolated cervical dystonia (CD). In addition to muscle relaxation at the peripheral level, few studies suggest that BoNT has effects on the central brain circuitries. The effects of BoNT on central circuitries that may be pain-related have not been examined. We probed these central effects with transcranial magnetic stimulation (TMS) techniques in a CD cohort presenting with significant pain.

**Methods:**

TMS-based measures of sensorimotor integration that are mediated through central processes, such as the short and long latency afferent inhibition (SAI and LAI) and measures for motor cortical excitability including short-interval intracortical inhibition (SICI) and intracortical facilitation (ICF) were recorded. These measures were recorded at specific interstimulus intervals (ISI) using paired-pulse paradigms before and after the peak effects of BoNT injections. Normative TMS data from age-matched healthy controls were collected for comparisons. Clinical pain symptoms were recorded with Toronto Western spasmodic rating scale (TWSTRS)-pain and a visual analog scale (VAS).

**Results:**

Eleven CD subjects (mean age ±SD, 53.1 ± 6.3 years) and 10 age-matched healthy controls were enrolled. SAI was found to be increased in CD patients at baseline, however at the time of peak BoNT effects, it revealed a significant change with normalization to healthy control data (SAI ISI 20 ms, *p* = 0.001; SAI ISI 30 ms, *p* = 0.03). The change in SAI correlated with improvements in pain levels assessed with TWSTRS-pain and VAS and the total dose of BoNT injected (corrected for multiple correlations). LAI, SICI, and ICF measures were similar to the healthy controls and remained unchanged with BoNT therapy.

**Conclusion:**

Pain control in CD from BoNT therapy relates to modulation of sensorimotor integration at the cortical level.

## Introduction

Cervical dystonia (CD) is a focal dystonia characterized by sustained muscle contractions that result in abnormal neck postures [1]. CD has one of the highest prevalence of pain, affecting more than a third of patients [2, 3]. Pain in CD is typically described as tightness, pulling, achiness, or knots in the neck and shoulder muscles [4]. Pain is a sensory symptom commonly related to the severity of dystonic postures and chronicity of the disease. Sometimes orthopedic complications such as spine degeneration, disk herniation, and radiculopathy that develop secondary to chronic CD symptoms further contribute to pain and disability [5].

Botulinum toxin (BoNT) injection therapy is a well-established therapy for alleviating pain in CD [6]. The commonly perceived mechanism of action for BoNT is peripheral muscle paralysis resulting from inhibition of acetylcholine release at the neuromuscular junction. However, there are an increasing number of preclinical studies that have shown that BoNT exhibits benefits beyond muscle relaxation effects and these are related to distinct central mechanisms [7, 8, 9, 10]. A few clinical studies using transcranial magnetic stimulation (TMS) techniques, primarily conducted in focal limb dystonia have also provided support for central effects resulting from peripheral BoNT injections [11, 12, 13, 14]. However, these studies have not examined these effects in the context of pain symptoms of dystonia.

We examined CD patients complaining of significant dystonia related pain with TMS techniques [15]. We examined the sensorimotor integration and motor cortex excitability that are core pathophysiological substrates for dystonia before and after the BoNT injections. We sought to understand whether the BoNT mediated pain control was related to the central changes measured with TMS.

## Methods

In an IRB approved protocol, CD patients receiving regular BoNT injections were enrolled from a movement disorders clinic at the University of Florida. Healthy controls were enrolled to acquire normative TMS data. Clinical diagnosis of CD was established using the guidelines laid by the movement disorders consensus committee [16]. All subjects enrolled in the study reported pain related to CD. Subjects enrolled did not have comorbidities to explain pain symptoms in the neck region (such as significant cervical spondylosis) as these could confound the assessment. Each CD subject was scheduled for two study visits. The first baseline visit was scheduled before the participants received the usual doses of BoNT injections. The baseline visit was scheduled at least 14 weeks after the last injection cycle when patients reported the benefits of previous injection cycle had worn off (BoNT_trough_). The second study visit was scheduled at approximately 4–6 weeks after injections once the CD subjects confirmed the achievement of usual peak benefits from BoNT therapy (BoNT_peak_). Oral medications for dystonia were withheld 12–24 h before each of the study visits. At each visit, the pain and motor severity associated with dystonia was measured with the Toronto Western spasmodic torticollis rating scale (TWSTRS). Additionally, the visual analogue scale (VAS) scale was administered for assessment of pain and dystonia related disability.

Electromyography (EMG) was recorded with a Bagnoli amplifier (Delsys, USA) from the first dorsal interosseous (FDI) muscle on the dominant side. We chose this muscle instead of the neck muscle as the TMS protocols have been standardized mainly for the hand muscles, and our goal was to determine the change occurring in response to BoNT injections. EMG signals captured via the Ag-AgCl surface electrodes were amplified (1K) and band-pass filtered (bandwidth from 20 Hz to 450 Hz). A CED 1401 device (Cambridge Electronic Design, UK) at a sampling rate of 5K was used for analog to digital conversion. EMG traces were analyzed using signal version 5.12.

TMS studies were conducted using standard protocols to assess the sensorimotor integration and the motor cortex excitability. TMS was applied to the primary motor cortex through Magstim 200 [2] stimulators with a monophasic current waveform (Magstim Company, UK). A figure-of-eight coil with an outer loop diameter of 7 cm was held tangentially over the cortex contralateral to the dominant hand. The coil pointed backward and laterally with a 45° angle to the sagittal plane. The “hot spot” was marked with a sharpie pen over the scalp when consistent motor evoked potentials (MEPs) of maximal amplitudes were recorded from the contralateral FDI muscle. Rest motor threshold (RMT) and active motor threshold (AMT) were recorded. RMT was defined as the minimum stimulus intensity that produced a MEP of 50 μV in 5 out of 10 consecutive trials. AMT was defined as the minimum stimulus intensity at which MEPs of 150 μV amplitude were elicited in tonically contracting FDI muscle (approximately 10% of maximum voluntary contraction). TS was defined as the intensity needed to evoke MEPs with peak-to-peak amplitudes of approximately 1 mV. Trials with background EMG activity appearing before delivery of any stimulus were rejected.

Short-latency afferent inhibition (SAI) at interstimulus intervals (ISI) of 20 and 30 ms and long-latency afferent inhibition (LAI) at ISI of 150 ms and 200 ms were recorded with paired-pulse protocols for assessment of sensorimotor integration [17]. In the paired-pulse protocol, median nerve stimulation on the dominant side preceded TMS pulse to the contralateral motor cortex. The ISI selected for SAI (20 ms and 30 ms) and LAI (150 ms and 200 ms) were based on previous studies that showed these ISI were effective in the elicitation of sensorimotor inhibition [18, 19]. The median nerve stimulation was applied to the dominant side wrist through a bipolar electrode (cathode proximal) using a constant current square wave pulse width duration of 200 μsec). The intensity for median nerve stimulation was kept just above threshold for evoking a slight twitch in the abductor pollicis brevis muscle.

Short interval intracortical inhibition (SICI) at an ISI of 2 ms and 3 ms and intracortical facilitation (ICF) at an ISI of 10 ms and at 15 ms were recorded for assessment of motor cortex excitability. In these protocols, paired pulses were delivered to the contralateral primary motor cortex in which the conditioning pulse preceded the test pulse. The intensity of the conditioning stimulus was set at 80% of AMT. The interval between conditioning pulse and test pulse, the interstimulus interval (ISI), was selected as 2 ms and 3 ms for SICI and 10 ms and 15 ms for ICF. In paired pulse trials, 10 for each ISI and TS alone were delivered in a randomized order for 90 trials. The inter-trial interval varied randomly between 4 and 6 s. Trials with a significant background EMG area about 500 ms before the TMS pulse were rejected. Peak-to-peak MEP amplitudes were averaged across all trials for TMS measures and were expressed as the ratio of conditioned (with preceding pulses) to the unconditioned (test pulse alone) MEP. Ratios <1 represented inhibition, and ratios >1 indicated facilitation. The mean peak-to-peak MEP amplitude was determined for TS trials, and for SAI, LAI, SICI and ICF trials. For the determination of SAI, LAI, SICI, and ICF, the mean peak-to-peak amplitude at each ISI (conditioned MEP) was expressed as a percentage of the mean peak-to-peak amplitude of the unconditioned test pulse (test MEP). Data were expressed as means ± standard deviation for demographics, disease-related characteristics, dose and duration of BoNT therapy, and clinical and TMS measures.

### Statistical analysis

Data were analyzed off-line using SPSS software version 26. Non-parametric tests were used if the values were not normally distributed (*p* < 0.05, Kolmogorov Smirov test). Significant SAI, LAI, SICI and ICF was determined by comparing the MEP amplitudes for the test stimulus preceded by conditioned stimulus with that of the test stimulus alone using the paired t-test. We determined whether the TMS measures in CD group (BoNT_trough_) significantly differed from the healthy controls using non-parametric one-way ANOVA test. We used two-way repeated measures ANOVA for SAI, LAI, SICI and ICF within the CD group to determine the effects and/or interactions between BoNT injections (BoNT_trough_ and BoNT_peak_) and ISI. If interaction effects were significant, *post hoc* tests with Bonferroni corrections were used. We used paired t-test to compare differences between BoNT_trough_ and BoNT_peak_ in the CD severity, pain scores and measures of motor cortex excitability (RMT, AMT). The level of statistical significance was set to *p* < 0.05. Using Spearman’s correlation test, we correlated the BoNT induced changes in TMS measures with clinical variables such as age, disease duration, disease severity, total BoNT dose, duration of BoNT therapy in years and the total number of muscles injected. We also correlated change in TMS measures with the motor and pain improvements in response to BoNT therapy. Bonferroni corrections were applied for multiple correlations (*p* < 0.01 significant).

## Results

Eleven CD patients (mean age ±SD, 53.1 ± 6.3 years) receiving BoNT every 12 weeks and ten age and gender matched healthy subjects (51.4 ± 5.7 years) were enrolled. All subjects except one received BoNT type A therapy (one received BoNT type B). Demographic data of the CD group and clinical characteristics pertaining to pain symptoms have been summarized in Table 1.

### Baseline comparison of CD with healthy controls

One-way ANOVA revealed a significantly greater inhibition in SAI (SAI 20, *p* = 0.04; SAI 30, *p* = 0.05) during BoN_Ttrough_ compared to healthy controls. However, LAI 150 (*p* = 0.53) and LAI 200 (*p* = 0.65) for BoNT_trough_ were not significantly different compared to the healthy controls. Similarly, SICI 2 (*p* = 0.27), SICI 3 (*p* = 0.67), ICF 10 (*p* = 0.37) and ICF 15 (*p* = 0.24) were not different from healthy controls.

### BoNT influence on TMS measures in CD

**FIGURE 1:**
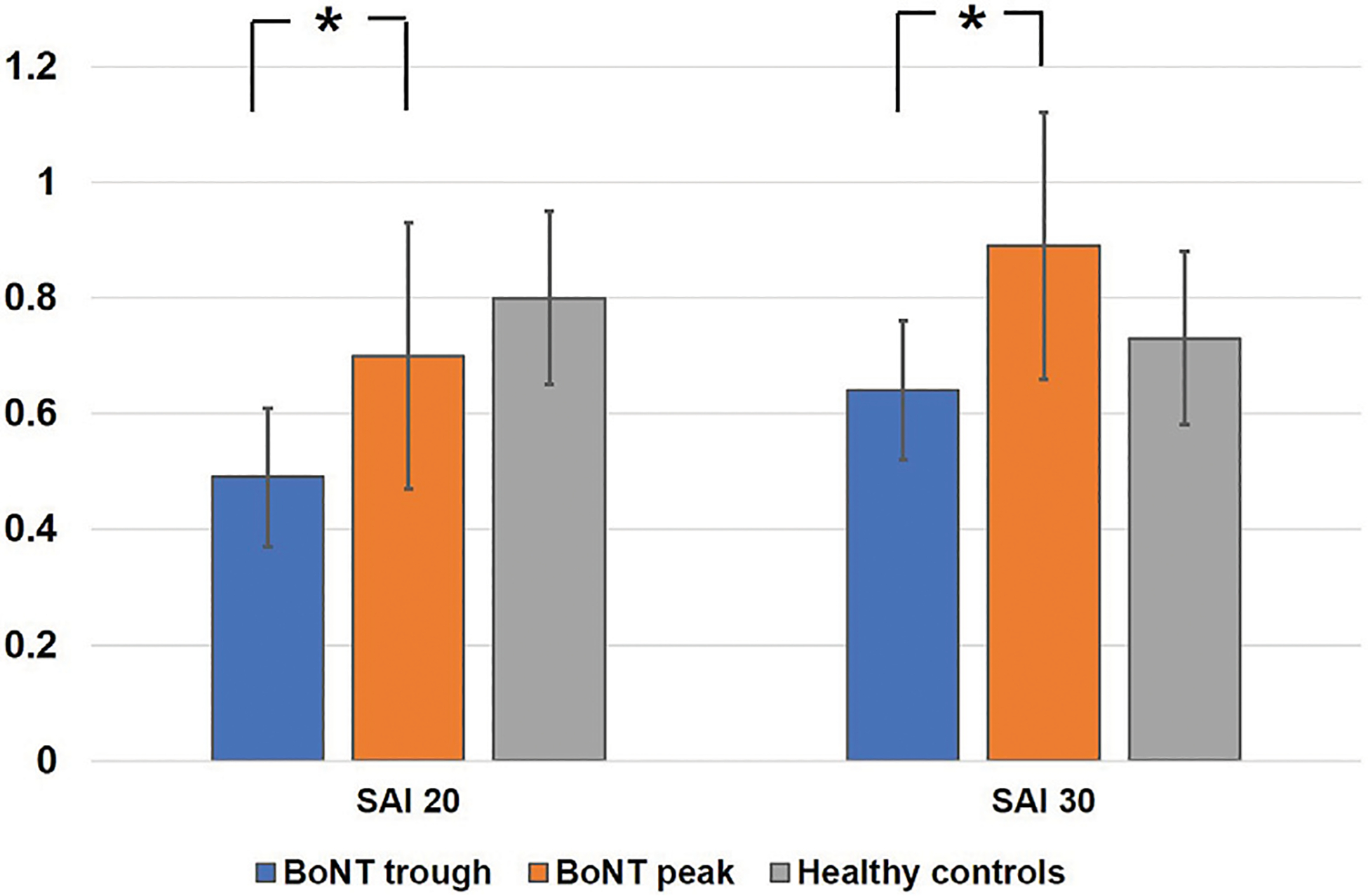
Represents mean scores of effects of botulinum toxin on short latency afferent inhibition at interstimulus intervals of 20 ms and 30 ms. The y-axis represents afferent inhibition as ratios of the conditioned (test stimulus with preceding median nerve stimulation) to the unconditioned (test stimulus alone) MEP amplitude. Values <1 indicate short latency afferent inhibition. White bars represent healthy controls, blue represent BoNT_trough_ and orange bars are BoNT_peak_. Error bars represent standard errors of mean. Asterisks between the bars illustrate significant differences between the groups on ANOVA and *post hoc* testing *p* < 0.05.

In the comparison between BoNT_trough_ and BoNT_peak_, the two-way repeated measures ANOVA revealed a significant main effect of BoNT (F_1,10_ = 36.3, *p* = 0.04) on the SAI measure and the main effect of ISI approached significance (F_1,10_ = 4.2, *p* = 0.06). The interaction effect of the two factors was significant (F_1,10_ = 3.10, *p* = 0.03). Post hoc analysis revealed significant difference between BoNT_trough_ and BoNT_peak_ (SAI 20, *p* = 0.001; SAI 30, *p* = 0.03). Figure 1. ANOVA for LAI showed no significant main effect of BoNT (F_1,10_ = 0.3, *p* = 0.59), ISI (F_1,10_ = 2, *p* = 0.18) or interaction between the two factors (F_1,10_ = 2.9, *p* = 0.12).

For SICI, the main effects of ISI approached significance (F_1,10_ = 3.7, *p* = 0.08) however there were no significant effects of BoNT (F_1,10_ = 0.59, *p* = 0.46) and there were no interactions between BoNT and ISI (F_1,10_ = 0.84, *p* = 0.38). Similarly, ANOVA for ICF showed no significant effect for ISI (F_1,10_ = 0.23, *p* = 0.64), BoNT group (F_1,10_ = .09, *p* = 0.76) and ISI and BoNT interaction (F_1,10_ = 1.9, *p* = 0.19). Paired t-test comparisons for BoNT_trough_ and BoNT_peak_ were not significant for RMT (54.3% ± 5.8% stimulator output, *p* = 0.68) and AMT (48.3% ± 6.5% stimulator output, *p* = 0.81).

Amongst the baseline factors such as age, disease duration, disease severity, pain level, duration of BoNT therapy, number of muscles injected and BoNT dose, only the total dose injected contributed significantly to the absolute change in SAI (SAI 20, *p* = 0.006, r = 0.45; SAI 30, *p* = 0.005, r = 0.38).

### BoNT effects on clinical measures in CD

The TWSTRS motor severity score was 17.3 (±7.3) at BoNT_trough_ which improved significantly when recorded at BoNT_peak_ (46% improvement, *p* = 0.004). Similarly, the VAS disability score of 6.1 (±2.5) at BoNT_trough_ improved significantly at BoNT_peak_ (59% improvement, *p* = 0.001). The mean TWSTRS pain score (BoNT_trough_ 8.0, BoNT_peak_ 1.7) improved by 75% (*p* = 0.004) and the VAS pain score (BoNT_trough_ 5.0, BoNT_peak_ 1.1) improved by 78% (*p* = 0.001) upon reaching BoNT_peak_.

### BoNT effects on sensorimotor integration correlates with pain improvement

**FIGURE 2:**
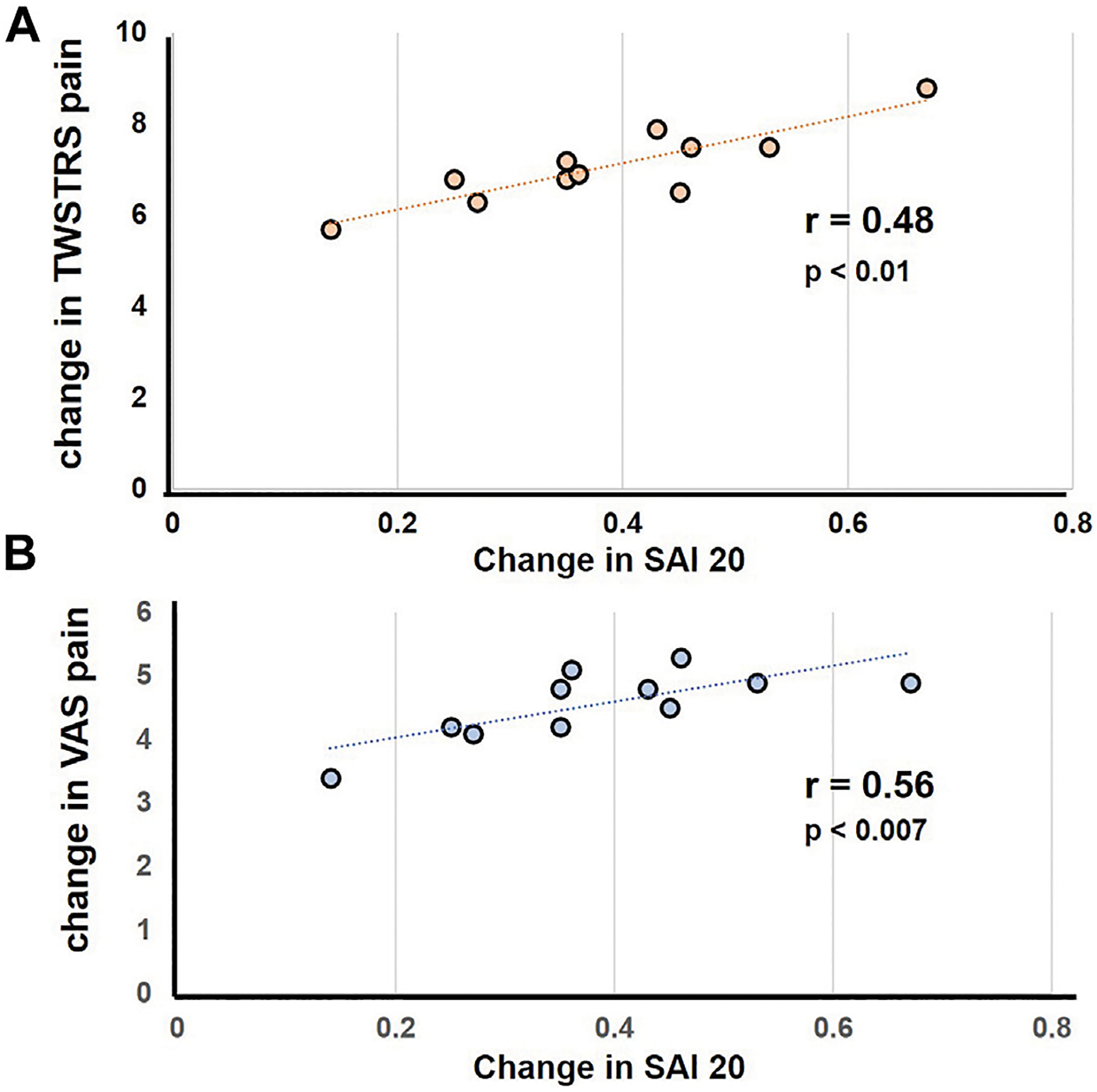
Represents correlation between the change in pain score in response to botulinum toxin injections with change in SAI. The scatter plot illustrates a significant linear correlation between decline in pain scores and decrease in SAI. **(A)** Represents correlation analysis for change in SAI 20 with change in TWSTRS **(B)** represents correlation analysis for change in SAI 20 with change in VAS. Each filled circle represents data for individual subjects.

The BoNT induced absolute change in SAI 20 (BoNT_trough_ 0.43, BoNT_peak_ 0.74) correlated with improvement in pain scores on the TWSTRS (r = 0.61, *p* = 0.001) and the VAS pain scale *p* = 0.01, r = 0.53); Figure 2. Similarly, the change in SAI 30 (BoNT_trough_ 0.67, BoNT_peak_ 0.91) correlated significantly with pain improvements on the TWSTRS scale (r = 0.48, *p* = 0.01) and the VAS scale (r = 0.56, *p* = 0.007). Table 2 Other correlations including correlations between change in motor scores and pain improvements were not statistically significant.

## Discussion

The main findings of the study are that BoNT therapy modulates sensorimotor integration in CD assessed with TMS-based SAI measure and these correlate with clinical pain improvements.

SAI is an inhibitory cortical phenomenon that is elicited when a peripheral sensory input interacts with the motor cortex through a short latency conduction pathway. Although several studies have indicated that sensory processing is abnormal in dystonia, only a few have examined specific cohorts such as focal dystonia [12]. SAI when examined in a writer’s cramp study was observed to increase in the abductor digiti minimi muscle during phasic contraction of the FDI muscle. This increase in SAI was speculated to prevent overflow of dystonia in surrounding muscles [20]. In our study, SAI recorded from the hand muscles was abnormal. Thus, SAI was found to be abnormal in body region that was asymptomatic which is not surprising, as prior dystonia research has shown physiological abnormalities to often affect the body regions that are clinically uninvolved [21]. Our study found that SAI was increased during BoNT_trough_ compared to healthy controls and was observed to reduce at BoNT_peak_. A previous study by Kojovic et al found SAI measure to be unaffected in CD regardless of whether the measurement was performed with and without botulinum toxin injections. Many patients in this cohort presented with segmental dystonia instead of a focal CD phenotype. More importantly it was not clear whether the participants complained of pain. Then there were other methodological differences. The ISI used for SAI measurement was 25 ms instead of 20 ms and 30 ms and the timing of BoNT_peak_ recording was 1 month after injections instead of a subjective peak of benefits endorsed by the patients in our study [14]. In another study by Zittel et al, SAI was observed to be reduced in patients with CD when measured at ISI of 25 ms, 30 ms, and 40 ms [22]; however, this study also did not focus on pain. SAI has been found to be associated with pain processing and pathway as demonstrated in a previous study involving patients with complex regional pain syndrome. Similar to our study, the participants in this study complained of significant neuropathic pain and had an increased SAI [23].

Although pain is common in CD, the underlying pathophysiological basis is less well understood. It is speculated that excessive muscle spasm, increased number of pain receptors in the neck muscles and a lower than normal pain threshold are likely important reasons [24] Pain may not always fully correlate with motor symptomatology or changes in posture. Pain is one of the first symptoms of CD to re-emerge as the effects of BoNT-A wear-off. The current available clinical scales do not capture the full extent or spectrum of pain description provided by the CD patients [3, 5]. The TWSTRS pain subscale comprises a severity score for the patient’s usual, worst, and best pain, a duration of pain component, as well as an assessment of disability due to pain without consideration to the location of pain. The CDIP-58 questionnaire includes five pain related items including aching shoulder, shoulder pain, tired neck and shoulder, tightness in neck and tightness in shoulder [25]. However, some patients with CD (10%–20%) complain of bioccipital headache [26] and these symptoms may not be captured by the TWSTRS and the CDIP-58 scale.

There are many possible explanations for why BoNT therapy led to modulation of the SAI. BoNT injected peripherally has been suggested to affect the inputs arising from the intrafusal afferent axons [27] which in turn modulates the amount of supraspinal sensory input reaching the motor cortex [7]. Central effects of BoNT-A may occur by indirect reorganization of sensorimotor integration involving spinal cord circuitry, brainstem, and the motor cortex [10]. In an animal study, radioactivity was found to spread to spinal cord segments contralateral to the injection and in smaller amounts to the dorsal roots [28]. BoNT therapy in CD has also been shown to possibly reverse the topography in the motor cortex which likely explains its effects on the cortical phenomenon of SAI (interaction of sensory input with the motor cortex) [18]. While a large number of patients recognize a muscle related pain [3], accumulating evidence suggests that non-muscle-based mechanisms (such as abnormal processing of nociceptive stimuli, abnormal transmission along the supraspinal afferent pathways dysfunction of descending pain inhibitory pathways as well as structural and network changes in the basal ganglia, cortex and other areas) may also contribute to pain. In one study, during the application of a conditioning stimulus, CD patients lacked the physiological reduction of pain perception and the nociceptive evoked potentials to laser stimuli [29]. The investigators speculated that abnormal inhibitory descending pain pathways might predispose patients with cervical dystonia to develop clinical pain [30]. The BoNT effects on neuropathic pain could be attributable to many factors including inhibition of pain mediator release, inhibition of membrane sodium channels, retrograde axonal transport and impact on the other pain pathways [31, 32] In our study, dystonia subjects reported pain relief upon receiving injections which correlated with the BoNT effects on SAI. However, based on the clinical descriptors provided by our patients, we could not ascertain whether the pain was related to peripheral or central factors. A significant correlation between BoNT related pain control and SAI modulation suggests that they likely share a common supraspinal pathway.

We acknowledge that our study has limitations. BoNT_trough_ may not represent a complete washout from the previous BoNT cycle in all subjects. Although additional data could have been collected at the end of the 12-week BoNT cycle, we anticipated no difference from data collected at the beginning of the cycle (BoNT_trough_). Detailed objective characterization of pain was not present. A control CD group that did not report pain manifestation was lacking. Multiple comparisons in a small sample may limit the generalizability of the results. Motor improvements in response to BoNT could impact the change in SAI and the pain scores; however, these were not accounted for in the analysis. Future research should include a detailed quantitative assessment of pain and muscle spasms to elucidate further the relationship between BoNT therapy, sensorimotor integration, and pain control.

## Figures and Tables

**TABLE 1:** Clinical profile and demographics of patients enrolled.

Patient	Age in yrs In years	Sex	Dystonia duration in yrs	Body distribution	Pain type	Treatment duration in yrs	BoNT dosages & number of muscles injected	TWSTRS severity score	TWSTRS pain score	VAS disability score	VAS pain score	Medications
1	58	F	7	Left torticollis	Aching	4	250, 7	10	11	5	1	Fluoxetine 20 mg
2	60	F	23	Left torticollis	Tightness neck & shoulder	10	350, 6	8	0	3	3	None
3	61	M	40	Right retrocolllis	Tightness shoulder	12	400, 10	24	11	5	3	Clonazepam 1 mg, Trazodone 50 mg
4	49	F	6	Mixed	Knots, tightness	3	250, 6	27	14	9	8	Baclofen 15 mg
5	57	F	6	Retrocollis	Pulling headaches	4	300, 6	11	3	5	6	Clonazepam 1 mg
6	63	F	9	Mixed	Aching burning	4	280, 7	13	6.5	2	2	Acetaminophen, butalbital, caffeine
7	71	M	11	Right torticollis	Pulling	4	275, 8	23	0	6	0	Gabapentin 600 mg, Primidone 100 mg
8	74	M	15	Left torticollis	Tightness neck	6	350, 9	22	15	8	6	Clonazepam 1 mg, Pramipexole 0.5 mg
9	58	M	3	Mixed	Aching	1	200, 4	23	8	8	5	None
10	62	F	5	Retrocollis	Headaches	4	280, 6	17	10.5	7	4	Clonazepam 1 mg
11	41	F	18	Mixed	Knots, tightness	5	10,000 U BoNT type B, 10	13	18	10	7	Primidone 100 mg

M, males; F, females; TWSTRS, Toronto Western spasmodic torticollis rating scale; VAS, Visual analogue scale; BoNT, botulinum toxin; yrs, years.

**TABLE 2:** Means of electrophysiological measures and their correlations with clinical measures.

	SAI 20	SAI 30	LAI 150	LAI 200	SICI 2	SICI 3	ICF 10	ICF 15
Means ± SD								
Healthy controls	0.74 ± 0.23	0.87 ± 0.35	0.80 ± 0.35	0.62 ± 0.27	0.74 ± 0.15	0.72 ± 0.30	1.31 ± 0.34	1.36 ± 0.33
BoNT OFF	0.47 ± 0.18	0.67 ± 0.21	0.97 ± 0.67	0.62 ± 0.47	1.11 ± 0.84	1.12 ± 0.96	1.41 ± 1.27	1.42 ± 1.57
BoNT ON	0.71 ± 0.19	0.91 ± 0.42	0.97 ± 0.69	0.67 ± 0.36	0.89 ± 0.21	0.79 ± 0.36	1.42 ± 1.28	1.43 ± 0.77
Variable correlations								
TWSTRS severity change	r = −0.42	r = −0.24	r = −0.16	r = −0.17	r = −0.19	r = −0.08	r = −0.26	r = −0.21
	*p* = 0.13	*p* = 0.34	*p* = 0.65	*p* = 0.58	*p* = 0.68	*p* = 0.8	*p* = 0.58	*p* = 0.51
TWSTRS Pain change	r = −0.61	r = −0.48	r = −0.14	r = −0.17	r = −0.06	r = −0.23	r = −0.28	r = −0.19
	***p* = 0.001**	***p* = 0.01**	*p* = 0.51	*p* = 0.58	*p* = 0.68	*p* = 0.67	*p* = 0.53	*p* = 0.56
VAS disability change	r = −0.16	r = −0.25	r = −0.19	r = −0.13	r = −0.03	r = −0.28	r = −0.31	r = −0.46
	*p* = 0.76	*p* = 0.46	*p* = 0.67	*p* = 0.68	*p* = 0.91	*p* = 0.61	*p* = 0.52	*p* = 0.11
VAS pain score change	r = −0.53	r = −0.56	r = −0.16	r = −0.09	r = −0.01	r = −0.21	r = −0.34	r = −0.39
	***p* = 0.01**	***p* = 0.007**	*p* = 0.87	*p* = 0.77	*p* = 0.93	*p* = 0.74	*p* = 0.59	*p* = 0.23
Age	r = 0.15	r = 0.23	r = 0.17	r = 0.32	r = 0.15	r = 0.31	r = 0.11	r = 0.18
	*p* = 0.45	*p* = 0.51	*p* = 0.79	*p* = 0.26	*p* = 0.66	*p* = 0.14	*p* = 0.78	*p* = 0.62
Disease duration	r = 0.19	r = 0.15	r = 0.16	r = 0.15	r = 0.23	r = 0.10	r = 0.31	r = 0.25
	*p* = 0.25	*p* = 0.28	*p* = 0.56	*p* = 0.45	*p* = 0.56	*p* = 0.71	*p* = 0.55	*p* = 0.45
Disease severity	r = 0.25	r = 0.16	r = 0.18	r = 0.31	r = 0.19	r = 0.21	r = 0.13	r = 0.21
	*p* = 0.31	*p* = 0.45	*p* = 0.47	*p* = 0.35	*p* = 0.61	*p* = 0.65	*p* = 0.74	*p* = 0.68
BoNT dose	r = −0.45	r = −0.38	r = −0.17	r = −0.25	r = −0.18	r = −0.14	r = −0.13	r = −0.15
	***p* = 0.008**	***p* = 0.056**	*p* = 0.57	*p* = 0.61	*p* = 0.56	*p* = 0.53	*p* = 0.45	*p* = 0.61
Duration of BoNT therapy	r = 0.15	r = 0.15	r = 0.15	r = 0.15	r = 0.15	r = 0.15	r = 0.15	r = 0.15
	*p* = 0.45	*p* = 0.45	*p* = 0.45	*p* = 0.45	*p* = 0.45	*p* = 0.45	*p* = 0.45	*p* = 0.45
Number of muscles injected	r = −0.34	r = −0.28	r = −0.15	r = −0.13	r = −0.15	r = −0.16	r = −0.21	r = −0.28
	*p* = 0.14	*p* = 0.26	*p* = 0.69	*p* = 0.71	*p* = 0.67	*p* = 0.57	*p* = 0.58	*p* = 0.61

Means ± standard deviation for all TMS measures are illustrated. Correlations between baseline measures and change in TMS measures and correlations between clinical score improvements and change induced in TMS measures are shown. Bold values represent the *p* values were significant.

## Data Availability

The raw data supporting the conclusion of this article will be made available by the authors, without undue reservation.
